# Supplementary material for: Resolving the full spectrum of human genome variation using Linked-Reads
Source: Genome Res. 2019 Apr;29(4):635–45. doi: 10.1101/gr.234443.118 (PMC6442396; doi:10.1101/gr.234443.118)
Supplement: Supplemental Material [file supp_gr.234443.118_Supplemental_longranger-2.2.zip › longranger-2.2.tar.bz2!/lr2.2-public/lib/stan/cmdstan/stan/doc/api/html/reader_8hpp_source.html]

Stan: src/stan/io/reader.hpp Source File


|  |  |
| --- | --- |
|  | Stan  2.5.0  probability, sampling & optimization |


- Main Page
- Related Pages
- Namespaces
- Classes
- Files

- File List
- File Members

All Classes Namespaces Files Functions Variables Typedefs Enumerations Enumerator Friends Macros Pages

- src
- stan
- io

reader.hpp

Go to the documentation of this file.

1 #ifndef STAN\_\_IO\_\_READER\_HPP

2 #define STAN\_\_IO\_\_READER\_HPP

3

4 #include <boost/throw\_exception.hpp>

5 #include <stan/prob/transform.hpp>

6

7 namespace stan {

8

9  namespace io {

10

11

33  template <typename T>

34  class reader {

35

36  private:

37

38  std::vector<T>& data\_r\_;

39  std::vector<int>& data\_i\_;

40  size\_t pos\_;

41  size\_t int\_pos\_;

42

43  inline T& scalar\_ptr() {

44  return data\_r\_.at(pos\_);

45  }

46

47  inline T& scalar\_ptr\_increment(size\_t m) {

48  pos\_ += m;

49  return data\_r\_.at(pos\_ - m);

50  }

51

52  inline int& int\_ptr() {

53  return data\_i\_.at(int\_pos\_);

54  }

55

56  inline int& int\_ptr\_increment(size\_t m) {

57  int\_pos\_ += m;

58  return data\_i\_.at(int\_pos\_ - m);

59  }

60

61  public:

62

63  typedef Eigen::Matrix<T,Eigen::Dynamic,Eigen::Dynamic> matrix\_t;

64  typedef Eigen::Matrix<T,Eigen::Dynamic,1> vector\_t;

65  typedef Eigen::Matrix<T,1,Eigen::Dynamic> row\_vector\_t;

66

67  typedef Eigen::Map<matrix\_t> map\_matrix\_t;

68  typedef Eigen::Map<vector\_t> map\_vector\_t;

69  typedef Eigen::Map<row\_vector\_t> map\_row\_vector\_t;

70

71

83  reader(std::vector<T>& data\_r,

84  std::vector<int>& data\_i)

85  : data\_r\_(data\_r),

86  data\_i\_(data\_i),

87  pos\_(0),

88  int\_pos\_(0) {

89  }

90

94  ~reader() { }

95

101  inline size\_t available() {

102  return data\_r\_.size() - pos\_;

103  }

104

110  inline size\_t available\_i() {

111  return data\_i\_.size() - int\_pos\_;

112  }

113

119  inline int integer() {

120  if (int\_pos\_ >= data\_i\_.size())

121  BOOST\_THROW\_EXCEPTION(

122  std::runtime\_error("no more integers to read."));

123  return data\_i\_[int\_pos\_++];

124  }

125

133  inline int integer\_constrain() {

134  return integer();

135  }

136

144  inline int integer\_constrain(T& /\*log\_prob\*/) {

145  return integer();

146  }

147

148

149

155  inline T scalar() {

156  if (pos\_ >= data\_r\_.size())

157  BOOST\_THROW\_EXCEPTION(std::runtime\_error("no more scalars to read"));

158  return data\_r\_[pos\_++];

159  }

160

167  inline T scalar\_constrain() {

168  return scalar();

169  }

170

182  T scalar\_constrain(T& /\*log\_prob\*/) {

183  return scalar();

184  }

185

186

194  inline std::vector<T> std\_vector(size\_t m) {

195  std::vector<T> vec;

196  T& start = scalar\_ptr\_increment(m);

197  vec.insert(vec.begin(), &start, &scalar\_ptr());

198  return vec;

199  }

200

208  inline vector\_t vector(size\_t m) {

209  return map\_vector\_t(&scalar\_ptr\_increment(m),m);

210  }

218  inline vector\_t vector\_constrain(size\_t m) {

219  return map\_vector\_t(&scalar\_ptr\_increment(m),m);

220  }

229  inline vector\_t vector\_constrain(size\_t m, T& /\*lp\*/) {

230  return map\_vector\_t(&scalar\_ptr\_increment(m),m);

231  }

232

233

234

242  inline row\_vector\_t row\_vector(size\_t m) {

243  return map\_row\_vector\_t(&scalar\_ptr\_increment(m),m);

244  }

245

253  inline row\_vector\_t row\_vector\_constrain(size\_t m) {

254  return map\_row\_vector\_t(&scalar\_ptr\_increment(m),m);

255  }

256

266  inline row\_vector\_t row\_vector\_constrain(size\_t m, T& /\*lp\*/) {

267  return map\_row\_vector\_t(&scalar\_ptr\_increment(m),m);

268  }

269

287  inline matrix\_t matrix(size\_t m, size\_t n) {

288  return map\_matrix\_t(&scalar\_ptr\_increment(m\*n),m,n);

289  }

290

301  inline matrix\_t matrix\_constrain(size\_t m, size\_t n) {

302  return map\_matrix\_t(&scalar\_ptr\_increment(m\*n),m,n);

303  }

304

317  inline matrix\_t matrix\_constrain(size\_t m, size\_t n, T& /\*lp\*/) {

318  return map\_matrix\_t(&scalar\_ptr\_increment(m\*n),m,n);

319  }

320

321

331  inline int integer\_lb(int lb) {

332  int i = integer();

333  if (!(i >= lb))

334  BOOST\_THROW\_EXCEPTION(

335  std::runtime\_error("required value greater than or equal to lb"));

336  return i;

337  }

347  inline int integer\_lb\_constrain(int lb) {

348  return integer\_lb(lb);

349  }

360  inline int integer\_lb\_constrain(int lb, T& /\*lp\*/) {

361  return integer\_lb(lb);

362  }

363

364

374  inline int integer\_ub(int ub) {

375  int i = integer();

376  if (!(i <= ub))

377  BOOST\_THROW\_EXCEPTION(

378  std::runtime\_error("required value less than or equal to ub"));

379  return i;

380  }

390  inline int integer\_ub\_constrain(int ub) {

391  return integer\_ub(ub);

392  }

403  int integer\_ub\_constrain(int ub, T& /\*lp\*/) {

404  return integer\_ub(ub);

405  }

406

419  inline int integer\_lub(int lb, int ub) {

420  // read first to make position deterministic [arbitrary choice]

421  int i = integer();

422  if (lb > ub)

423  BOOST\_THROW\_EXCEPTION(

424  std::runtime\_error("lower bound must be less than or equal to ub"));

425  if (!(i >= lb))

426  BOOST\_THROW\_EXCEPTION(

427  std::runtime\_error("required value greater than or equal to lb"));

428  if (!(i <= ub))

429  BOOST\_THROW\_EXCEPTION(

430  std::runtime\_error("required value less than or equal to ub"));

431  return i;

432  }

443  inline int integer\_lub\_constrain(int lb, int ub) {

444  return integer\_lub(lb,ub);

445  }

457  inline int integer\_lub\_constrain(int lb, int ub, T& /\*lp\*/) {

458  return integer\_lub(lb,ub);

459  }

460

461

462

472  inline T scalar\_pos() {

473  T x(scalar());

474  stan::math::check\_positive("stan::io::scalar\_pos(%1%)", x,

475  "Constrained scalar", (double\*)0);

476  return x;

477  }

478

486  inline T scalar\_pos\_constrain() {

487  return stan::prob::positive\_constrain(scalar());

488  }

489

500  inline T scalar\_pos\_constrain(T& lp) {

501  return stan::prob::positive\_constrain(scalar(),lp);

502  }

503

516  template <typename TL>

517  inline T scalar\_lb(const TL lb) {

518  T x(scalar());

519  stan::math::check\_greater\_or\_equal("stan::io::scalar\_lb(%1%)",

520  x, lb, "Constrained scalar",

521  (double\*)0);

522  return x;

523  }

524

536  template <typename TL>

537  inline T scalar\_lb\_constrain(const TL lb) {

538  return stan::prob::lb\_constrain(scalar(),lb);

539  }

540

552  template <typename TL>

553  inline T scalar\_lb\_constrain(const TL lb, T& lp) {

554  return stan::prob::lb\_constrain(scalar(),lb,lp);

555  }

556

557

558

571  template <typename TU>

572  inline T scalar\_ub(TU ub) {

573  T x(scalar());

574  stan::math::check\_less\_or\_equal("stan::io::scalar\_ub(%1%)", x, ub,

575  "Constrained scalar", (double\*)0);

576  return x;

577  }

578

590  template <typename TU>

591  inline T scalar\_ub\_constrain(const TU ub) {

592  return stan::prob::ub\_constrain(scalar(),ub);

593  }

594

606  template <typename TU>

607  inline T scalar\_ub\_constrain(const TU ub, T& lp) {

608  return stan::prob::ub\_constrain(scalar(),ub,lp);

609  }

610

625  template <typename TL, typename TU>

626  inline T scalar\_lub(const TL lb, const TU ub) {

627  T x(scalar());

628  stan::math::check\_bounded<T,TL,TU,T>

629  ("stan::io::scalar\_lub(%1%)", x, lb, ub, "Constrained scalar",0);

630  return x;

631  }

632

646  template <typename TL, typename TU>

647  inline T scalar\_lub\_constrain(const TL lb, const TU ub) {

648  return stan::prob::lub\_constrain(scalar(),lb,ub);

649  }

650

664  template <typename TL, typename TU>

665  inline T scalar\_lub\_constrain(TL lb, TU ub, T& lp) {

666  return stan::prob::lub\_constrain(scalar(),lb,ub,lp);

667  }

668

677  inline T prob() {

678  T x(scalar());

679  stan::math::check\_bounded<T,double,double,double>

680  ("stan::io::prob(%1%)", x, 0, 1, "Constrained probability", 0);

681  return x;

682  }

683

692  inline T prob\_constrain() {

693  return stan::prob::prob\_constrain(scalar());

694  }

695

706  inline T prob\_constrain(T& lp) {

707  return stan::prob::prob\_constrain(scalar(),lp);

708  }

709

710

711

712

724  inline T corr() {

725  T x(scalar());

726  stan::math::check\_bounded<T,double,double,double>

727  ("stan::io::corr(%1%)", x, -1, 1, "Correlation value",0);

728  return x;

729  }

730

739  inline T corr\_constrain() {

740  return stan::prob::corr\_constrain(scalar());

741  }

742

754  inline T corr\_constrain(T& lp) {

755  return stan::prob::corr\_constrain(scalar(),lp);

756  }

757

768  inline vector\_t unit\_vector(size\_t k) {

769  vector\_t theta(vector(k));

770  stan::math::check\_unit\_vector("stan::io::unit\_vector(%1%)", theta, "Constrained vector",

771  (double\*)0);

772  return theta;

773  }

774

785  inline

786  Eigen::Matrix<T,Eigen::Dynamic,1> unit\_vector\_constrain(size\_t k) {

787  return stan::prob::unit\_vector\_constrain(vector(k-1));

788  }

789

802  inline vector\_t unit\_vector\_constrain(size\_t k, T& lp) {

803  return stan::prob::unit\_vector\_constrain(vector(k-1),lp);

804  }

805

816  inline vector\_t simplex(size\_t k) {

817  vector\_t theta(vector(k));

818  stan::math::check\_simplex("stan::io::simplex(%1%)", theta, "Constrained vector",

819  (double\*)0);

820  return theta;

821  }

822

833  inline

834  Eigen::Matrix<T,Eigen::Dynamic,1> simplex\_constrain(size\_t k) {

835  return stan::prob::simplex\_constrain(vector(k-1));

836  }

837

850  inline vector\_t simplex\_constrain(size\_t k, T& lp) {

851  return stan::prob::simplex\_constrain(vector(k-1),lp);

852  }

853

864  inline vector\_t ordered(size\_t k) {

865  vector\_t x(vector(k));

866  stan::math::check\_ordered("stan::io::ordered(%1%)", x, "Constrained vector");

867  return x;

868  }

869

879  inline vector\_t ordered\_constrain(size\_t k) {

880  return stan::prob::ordered\_constrain(vector(k));

881  }

882

894  inline vector\_t ordered\_constrain(size\_t k, T& lp) {

895  return stan::prob::ordered\_constrain(vector(k),lp);

896  }

897

908  inline vector\_t positive\_ordered(size\_t k) {

909  vector\_t x(vector(k));

910  stan::math::check\_positive\_ordered("stan::io::positive\_ordered(%1%)",

911  x, "Constrained vector",

912  (double\*)0);

913  return x;

914  }

915

925  inline vector\_t positive\_ordered\_constrain(size\_t k) {

926  return stan::prob::positive\_ordered\_constrain(vector(k));

927  }

928

940  inline vector\_t positive\_ordered\_constrain(size\_t k, T& lp) {

941  return stan::prob::positive\_ordered\_constrain(vector(k),lp);

942  }

943

944

945

956  inline matrix\_t cholesky\_factor(size\_t M, size\_t N) {

957  matrix\_t y(matrix(M,N));

958  stan::math::check\_cholesky\_factor("stan::io::cholesky\_factor(%1%)",

959  y, "Constrained matrix",

960  (double\*)0);

961  return y;

962  }

963

975  inline matrix\_t cholesky\_factor\_constrain(size\_t M, size\_t N) {

976  return stan::prob::cholesky\_factor\_constrain(vector((N \* (N + 1)) / 2 + (M - N) \* N),

977  M,N);

978  }

979

993  inline matrix\_t cholesky\_factor\_constrain(size\_t M, size\_t N, T& lp) {

994  return stan::prob::cholesky\_factor\_constrain(vector((N \* (N + 1)) / 2 + (M - N) \* N),

995  M,N,lp);

996  }

997

998

999

1010  inline matrix\_t cholesky\_corr(size\_t K) {

1011  matrix\_t y(matrix(K,K));

1012  stan::math::check\_cholesky\_factor\_corr("stan::io::cholesky\_factor\_corr(%1%)", y, "Constrained matrix");

1013  return y;

1014  }

1015

1026  inline matrix\_t cholesky\_corr\_constrain(size\_t K) {

1027  return stan::prob::cholesky\_corr\_constrain(vector((K \* (K - 1)) / 2),

1028  K);

1029  }

1030

1043  inline matrix\_t cholesky\_corr\_constrain(size\_t K, T& lp) {

1044  return stan::prob::cholesky\_corr\_constrain(vector((K \* (K - 1)) / 2),

1045  K,lp);

1046  }

1047

1048

1049

1061  inline matrix\_t cov\_matrix(size\_t k) {

1062  matrix\_t y(matrix(k,k));

1063  stan::math::check\_cov\_matrix("stan::io::cov\_matrix(%1%)", y, "Constrained matrix",

1064  (double\*)0);

1065  return y;

1066  }

1067

1076  inline matrix\_t cov\_matrix\_constrain(size\_t k) {

1077  return stan::prob::cov\_matrix\_constrain(vector(k + (k \* (k - 1)) / 2),

1078  k);

1079  }

1080

1092  inline matrix\_t cov\_matrix\_constrain(size\_t k, T& lp) {

1093  return stan::prob::cov\_matrix\_constrain(vector(k + (k \* (k - 1)) / 2),

1094  k,lp);

1095  }

1096

1097

1107  inline matrix\_t corr\_matrix(size\_t k) {

1108  matrix\_t x(matrix(k,k));

1109  stan::math::check\_corr\_matrix("stan::math::corr\_matrix(%1%)",

1110  x, "Constrained matrix",

1111  (double\*)0);

1112  return x;

1113  }

1114

1123  inline matrix\_t corr\_matrix\_constrain(size\_t k) {

1124  return stan::prob::corr\_matrix\_constrain(vector((k \* (k - 1)) / 2),k);

1125  }

1126

1138  inline matrix\_t corr\_matrix\_constrain(size\_t k, T& lp) {

1139  return stan::prob::corr\_matrix\_constrain(vector((k \* (k - 1)) / 2),

1140  k,lp);

1141  }

1142

1143  template <typename TL>

1144  inline vector\_t vector\_lb(const TL lb, size\_t m) {

1145  vector\_t v(m);

1146  for (size\_t i = 0; i < m; ++i)

1147  v(i) = scalar\_lb(lb);

1148  return v;

1149  }

1150  template <typename TL>

1151  inline vector\_t vector\_lb\_constrain(const TL lb, size\_t m) {

1152  vector\_t v(m);

1153  for (size\_t i = 0; i < m; ++i)

1154  v(i) = scalar\_lb\_constrain(lb);

1155  return v;

1156  }

1157  template <typename TL>

1158  inline vector\_t vector\_lb\_constrain(const TL lb, size\_t m, T& lp) {

1159  vector\_t v(m);

1160  for (size\_t i = 0; i < m; ++i)

1161  v(i) = scalar\_lb\_constrain(lb,lp);

1162  return v;

1163  }

1164

1165  template <typename TL>

1166  inline row\_vector\_t row\_vector\_lb(const TL lb, size\_t m) {

1167  row\_vector\_t v(m);

1168  for (size\_t i = 0; i < m; ++i)

1169  v(i) = scalar\_lb(lb);

1170  return v;

1171  }

1172  template <typename TL>

1173  inline row\_vector\_t row\_vector\_lb\_constrain(const TL lb, size\_t m) {

1174  row\_vector\_t v(m);

1175  for (size\_t i = 0; i < m; ++i)

1176  v(i) = scalar\_lb\_constrain(lb);

1177  return v;

1178  }

1179  template <typename TL>

1180  inline row\_vector\_t row\_vector\_lb\_constrain(const TL lb, size\_t m, T& lp) {

1181  row\_vector\_t v(m);

1182  for (size\_t i = 0; i < m; ++i)

1183  v(i) = scalar\_lb\_constrain(lb,lp);

1184  return v;

1185  }

1186

1187  template <typename TL>

1188  inline matrix\_t matrix\_lb(const TL lb, size\_t m, size\_t n) {

1189  matrix\_t v(m,n);

1190  for (size\_t j = 0; j < n; ++j)

1191  for (size\_t i = 0; i < m; ++i)

1192  v(i,j) = scalar\_lb(lb);

1193  return v;

1194  }

1195  template <typename TL>

1196  inline matrix\_t matrix\_lb\_constrain(const TL lb, size\_t m, size\_t n) {

1197  matrix\_t v(m,n);

1198  for (size\_t j = 0; j < n; ++j)

1199  for (size\_t i = 0; i < m; ++i)

1200  v(i,j) = scalar\_lb\_constrain(lb);

1201  return v;

1202  }

1203  template <typename TL>

1204  inline matrix\_t matrix\_lb\_constrain(const TL lb, size\_t m, size\_t n, T& lp) {

1205  matrix\_t v(m,n);

1206  for (size\_t j = 0; j < n; ++j)

1207  for (size\_t i = 0; i < m; ++i)

1208  v(i,j) = scalar\_lb\_constrain(lb,lp);

1209  return v;

1210  }

1211

1212  template <typename TU>

1213  inline vector\_t vector\_ub(const TU ub, size\_t m) {

1214  vector\_t v(m);

1215  for (size\_t i = 0; i < m; ++i)

1216  v(i) = scalar\_ub(ub);

1217  return v;

1218  }

1219  template <typename TU>

1220  inline vector\_t vector\_ub\_constrain(const TU ub, size\_t m) {

1221  vector\_t v(m);

1222  for (size\_t i = 0; i < m; ++i)

1223  v(i) = scalar\_ub\_constrain(ub);

1224  return v;

1225  }

1226  template <typename TU>

1227  inline vector\_t vector\_ub\_constrain(const TU ub, size\_t m, T& lp) {

1228  vector\_t v(m);

1229  for (size\_t i = 0; i < m; ++i)

1230  v(i) = scalar\_ub\_constrain(ub,lp);

1231  return v;

1232  }

1233

1234  template <typename TU>

1235  inline row\_vector\_t row\_vector\_ub(const TU ub, size\_t m) {

1236  row\_vector\_t v(m);

1237  for (size\_t i = 0; i < m; ++i)

1238  v(i) = scalar\_ub(ub);

1239  return v;

1240  }

1241  template <typename TU>

1242  inline row\_vector\_t row\_vector\_ub\_constrain(const TU ub, size\_t m) {

1243  row\_vector\_t v(m);

1244  for (size\_t i = 0; i < m; ++i)

1245  v(i) = scalar\_ub\_constrain(ub);

1246  return v;

1247  }

1248  template <typename TU>

1249  inline row\_vector\_t row\_vector\_ub\_constrain(const TU ub, size\_t m, T& lp) {

1250  row\_vector\_t v(m);

1251  for (size\_t i = 0; i < m; ++i)

1252  v(i) = scalar\_ub\_constrain(ub,lp);

1253  return v;

1254  }

1255

1256  template <typename TU>

1257  inline matrix\_t matrix\_ub(const TU ub, size\_t m, size\_t n) {

1258  matrix\_t v(m,n);

1259  for (size\_t j = 0; j < n; ++j)

1260  for (size\_t i = 0; i < m; ++i)

1261  v(i,j) = scalar\_ub(ub);

1262  return v;

1263  }

1264  template <typename TU>

1265  inline matrix\_t matrix\_ub\_constrain(const TU ub, size\_t m, size\_t n) {

1266  matrix\_t v(m,n);

1267  for (size\_t j = 0; j < n; ++j)

1268  for (size\_t i = 0; i < m; ++i)

1269  v(i,j) = scalar\_ub\_constrain(ub);

1270  return v;

1271  }

1272  template <typename TU>

1273  inline matrix\_t matrix\_ub\_constrain(const TU ub, size\_t m, size\_t n, T& lp) {

1274  matrix\_t v(m,n);

1275  for (size\_t j = 0; j < n; ++j)

1276  for (size\_t i = 0; i < m; ++i)

1277  v(i,j) = scalar\_ub\_constrain(ub,lp);

1278  return v;

1279  }

1280

1281

1282  template <typename TL, typename TU>

1283  inline vector\_t vector\_lub(const TL lb, const TU ub, size\_t m) {

1284  vector\_t v(m);

1285  for (size\_t i = 0; i < m; ++i)

1286  v(i) = scalar\_lub(lb,ub);

1287  return v;

1288  }

1289  template <typename TL, typename TU>

1290  inline vector\_t vector\_lub\_constrain(const TL lb, const TU ub, size\_t m) {

1291  vector\_t v(m);

1292  for (size\_t i = 0; i < m; ++i)

1293  v(i) = scalar\_lub\_constrain(lb,ub);

1294  return v;

1295  }

1296  template <typename TL, typename TU>

1297  inline vector\_t vector\_lub\_constrain(const TL lb, const TU ub, size\_t m, T& lp) {

1298  vector\_t v(m);

1299  for (size\_t i = 0; i < m; ++i)

1300  v(i) = scalar\_lub\_constrain(lb,ub,lp);

1301  return v;

1302  }

1303

1304  template <typename TL, typename TU>

1305  inline row\_vector\_t row\_vector\_lub(const TL lb, const TU ub, size\_t m) {

1306  row\_vector\_t v(m);

1307  for (size\_t i = 0; i < m; ++i)

1308  v(i) = scalar\_lub(lb,ub);

1309  return v;

1310  }

1311  template <typename TL, typename TU>

1312  inline row\_vector\_t row\_vector\_lub\_constrain(const TL lb, const TU ub, size\_t m) {

1313  row\_vector\_t v(m);

1314  for (size\_t i = 0; i < m; ++i)

1315  v(i) = scalar\_lub\_constrain(lb,ub);

1316  return v;

1317  }

1318  template <typename TL, typename TU>

1319  inline row\_vector\_t row\_vector\_lub\_constrain(const TL lb, const TU ub, size\_t m, T& lp) {

1320  row\_vector\_t v(m);

1321  for (size\_t i = 0; i < m; ++i)

1322  v(i) = scalar\_lub\_constrain(lb,ub,lp);

1323  return v;

1324  }

1325

1326  template <typename TL, typename TU>

1327  inline matrix\_t matrix\_lub(const TL lb, const TU ub, size\_t m, size\_t n) {

1328  matrix\_t v(m,n);

1329  for (size\_t j = 0; j < n; ++j)

1330  for (size\_t i = 0; i < m; ++i)

1331  v(i,j) = scalar\_lub(lb,ub);

1332  return v;

1333  }

1334  template <typename TL, typename TU>

1335  inline matrix\_t matrix\_lub\_constrain(const TL lb, const TU ub, size\_t m, size\_t n) {

1336  matrix\_t v(m,n);

1337  for (size\_t j = 0; j < n; ++j)

1338  for (size\_t i = 0; i < m; ++i)

1339  v(i,j) = scalar\_lub\_constrain(lb,ub);

1340  return v;

1341  }

1342  template <typename TL, typename TU>

1343  inline matrix\_t matrix\_lub\_constrain(const TL lb, const TU ub, size\_t m, size\_t n, T& lp) {

1344  matrix\_t v(m,n);

1345  for (size\_t j = 0; j < n; ++j)

1346  for (size\_t i = 0; i < m; ++i)

1347  v(i,j) = scalar\_lub\_constrain(lb,ub,lp);

1348  return v;

1349  }

1350

1351

1352

1353  };

1354

1355  }

1356

1357 }

1358

1359 #endif

stan::math::check\_cov\_matrix

bool check\_cov\_matrix(const char \*function, const Eigen::Matrix< T\_y, Eigen::Dynamic, Eigen::Dynamic > &y, const char \*name, T\_result \*result)

Return true if the specified matrix is a valid covariance matrix.

**Definition:** check\_cov\_matrix.hpp:29

stan::io::reader::cholesky\_corr\_constrain

matrix\_t cholesky\_corr\_constrain(size\_t K, T &lp)

Return the next Cholesky factor for a correlation matrix with the specified dimensionality, reading from an unconstrained vector of the appropriate size, and increment the log probability reference with the log Jacobian adjustment for the transform.

**Definition:** reader.hpp:1043

stan::io::reader::positive\_ordered\_constrain

vector\_t positive\_ordered\_constrain(size\_t k)

Return the next positive ordered vector of the specified length.

**Definition:** reader.hpp:925

stan::io::reader::row\_vector\_ub\_constrain

row\_vector\_t row\_vector\_ub\_constrain(const TU ub, size\_t m)

**Definition:** reader.hpp:1242

stan::io::reader::simplex\_constrain

vector\_t simplex\_constrain(size\_t k, T &lp)

Return the next simplex of the specified size (using one fewer unconstrained scalars), incrementing the specified reference with the log absolute Jacobian determinant.

**Definition:** reader.hpp:850

stan::io::reader::scalar\_lub\_constrain

T scalar\_lub\_constrain(const TL lb, const TU ub)

Return the next scalar transformed to be between the specified lower and upper bounds.

**Definition:** reader.hpp:647

stan::io::reader::matrix\_lub

matrix\_t matrix\_lub(const TL lb, const TU ub, size\_t m, size\_t n)

**Definition:** reader.hpp:1327

stan::io::reader::scalar\_ub

T scalar\_ub(TU ub)

Return the next scalar, checking that it is less than or equal to the specified upper bound...

**Definition:** reader.hpp:572

stan::math::check\_ordered

bool check\_ordered(const char \*function, const Eigen::Matrix< T\_y, Eigen::Dynamic, 1 > &y, const char \*name, T\_result \*result)

Return true if the specified vector is sorted into increasing order.

**Definition:** check\_ordered.hpp:29

stan::prob::corr\_constrain

T corr\_constrain(const T x)

Return the result of transforming the specified scalar to have a valid correlation value between -1 a...

**Definition:** transform.hpp:952

stan::io::reader::scalar\_pos

T scalar\_pos()

Return the next scalar, checking that it is positive.

**Definition:** reader.hpp:472

stan::io::reader::unit\_vector\_constrain

vector\_t unit\_vector\_constrain(size\_t k, T &lp)

Return the next unit\_vector of the specified size (using one fewer unconstrained scalars), incrementing the specified reference with the log absolute Jacobian determinant.

**Definition:** reader.hpp:802

stan::io::reader::integer\_ub\_constrain

int integer\_ub\_constrain(int ub)

Return the next integer, checking that it is less than or equal to the specified upper bound...

**Definition:** reader.hpp:390

stan::io::reader::matrix\_ub

matrix\_t matrix\_ub(const TU ub, size\_t m, size\_t n)

**Definition:** reader.hpp:1257

stan::io::reader::matrix\_lub\_constrain

matrix\_t matrix\_lub\_constrain(const TL lb, const TU ub, size\_t m, size\_t n)

**Definition:** reader.hpp:1335

stan::io::reader::std\_vector

std::vector< T > std\_vector(size\_t m)

Return a standard library vector of the specified dimensionality made up of the next scalars...

**Definition:** reader.hpp:194

stan::io::reader::scalar\_lb

T scalar\_lb(const TL lb)

Return the next scalar, checking that it is greater than or equal to the specified lower bound...

**Definition:** reader.hpp:517

stan::io::reader::row\_vector\_constrain

row\_vector\_t row\_vector\_constrain(size\_t m)

Return a row vector of specified dimensionality made up of the next scalars.

**Definition:** reader.hpp:253

stan::io::reader::integer\_ub\_constrain

int integer\_ub\_constrain(int ub, T &)

Return the next integer, checking that it is less than or equal to the specified upper bound...

**Definition:** reader.hpp:403

stan::io::reader::scalar\_constrain

T scalar\_constrain()

Return the next scalar.

**Definition:** reader.hpp:167

stan::prob::cholesky\_factor\_constrain

Eigen::Matrix< T, Eigen::Dynamic, Eigen::Dynamic > cholesky\_factor\_constrain(const Eigen::Matrix< T, Eigen::Dynamic, 1 > &x, int M, int N)

Return the Cholesky factor of the specified size read from the specified vector.

**Definition:** transform.hpp:1412

stan::io::reader::vector\_lub\_constrain

vector\_t vector\_lub\_constrain(const TL lb, const TU ub, size\_t m, T &lp)

**Definition:** reader.hpp:1297

stan::io::reader::map\_row\_vector\_t

Eigen::Map< row\_vector\_t > map\_row\_vector\_t

**Definition:** reader.hpp:69

stan::io::reader::integer\_lb\_constrain

int integer\_lb\_constrain(int lb, T &)

Return the next integer, checking that it is greater than or equal to the specified lower bound...

**Definition:** reader.hpp:360

stan::io::reader::vector\_constrain

vector\_t vector\_constrain(size\_t m)

Return a column vector of specified dimensionality made up of the next scalars.

**Definition:** reader.hpp:218

stan::io::reader::row\_vector\_ub

row\_vector\_t row\_vector\_ub(const TU ub, size\_t m)

**Definition:** reader.hpp:1235

stan::io::reader::cov\_matrix

matrix\_t cov\_matrix(size\_t k)

Return the next covariance matrix with the specified dimensionality.

**Definition:** reader.hpp:1061

stan::prob::ordered\_constrain

Eigen::Matrix< T, Eigen::Dynamic, 1 > ordered\_constrain(const Eigen::Matrix< T, Eigen::Dynamic, 1 > &x)

Return an increasing ordered vector derived from the specified free vector.

**Definition:** transform.hpp:1224

stan::io::reader::matrix\_lb\_constrain

matrix\_t matrix\_lb\_constrain(const TL lb, size\_t m, size\_t n, T &lp)

**Definition:** reader.hpp:1204

stan::io::reader::corr\_constrain

T corr\_constrain()

Return the next scalar transformed to be a correlation between -1 and 1.

**Definition:** reader.hpp:739

stan::math::check\_simplex

bool check\_simplex(const char \*function, const Eigen::Matrix< T\_prob, Eigen::Dynamic, 1 > &theta, const char \*name, T\_result \*result)

Return true if the specified vector is simplex.

**Definition:** check\_simplex.hpp:32

stan::io::reader::scalar\_constrain

T scalar\_constrain(T &)

Return the next scalar in the sequence, incrementing the specified reference with the log absolute Ja...

**Definition:** reader.hpp:182

stan::io::reader::cov\_matrix\_constrain

matrix\_t cov\_matrix\_constrain(size\_t k)

Return the next covariance matrix of the specified dimensionality.

**Definition:** reader.hpp:1076

stan::io::reader::prob\_constrain

T prob\_constrain(T &lp)

Return the next scalar transformed to be a probability between 0 and 1, incrementing the specified re...

**Definition:** reader.hpp:706

stan::io::reader::corr\_matrix\_constrain

matrix\_t corr\_matrix\_constrain(size\_t k, T &lp)

Return the next correlation matrix of the specified dimensionality, incrementing the specified refere...

**Definition:** reader.hpp:1138

stan::io::reader::integer\_constrain

int integer\_constrain(T &)

Return the next integer in the integer sequence.

**Definition:** reader.hpp:144

stan::prob::simplex\_constrain

Eigen::Matrix< T, Eigen::Dynamic, 1 > simplex\_constrain(const Eigen::Matrix< T, Eigen::Dynamic, 1 > &y)

Return the simplex corresponding to the specified free vector.

**Definition:** transform.hpp:1103

stan::io::reader::corr

T corr()

Return the next scalar, checking that it is a valid value for a correlation, between -1 (inclusive) a...

**Definition:** reader.hpp:724

stan::io::reader::integer\_ub

int integer\_ub(int ub)

Return the next integer, checking that it is less than or equal to the specified upper bound...

**Definition:** reader.hpp:374

stan::io::reader::integer\_lb\_constrain

int integer\_lb\_constrain(int lb)

Return the next integer, checking that it is greater than or equal to the specified lower bound...

**Definition:** reader.hpp:347

stan::io::reader::scalar\_lb\_constrain

T scalar\_lb\_constrain(const TL lb)

Return the next scalar transformed to have the specified lower bound.

**Definition:** reader.hpp:537

stan::io::reader::cholesky\_corr

matrix\_t cholesky\_corr(size\_t K)

Return the next Cholesky factor for a correlation matrix with the specified dimensionality, reading it directly without transforms.

**Definition:** reader.hpp:1010

stan::io::reader::scalar\_pos\_constrain

T scalar\_pos\_constrain()

Return the next scalar, transformed to be positive.

**Definition:** reader.hpp:486

stan::io::reader::vector\_t

Eigen::Matrix< T, Eigen::Dynamic, 1 > vector\_t

**Definition:** reader.hpp:64

stan::io::reader::vector\_ub

vector\_t vector\_ub(const TU ub, size\_t m)

**Definition:** reader.hpp:1213

stan::io::reader::row\_vector

row\_vector\_t row\_vector(size\_t m)

Return a row vector of specified dimensionality made up of the next scalars.

**Definition:** reader.hpp:242

stan::io::reader::vector

vector\_t vector(size\_t m)

Return a column vector of specified dimensionality made up of the next scalars.

**Definition:** reader.hpp:208

stan::io::reader::vector\_lub

vector\_t vector\_lub(const TL lb, const TU ub, size\_t m)

**Definition:** reader.hpp:1283

stan::io::reader::row\_vector\_constrain

row\_vector\_t row\_vector\_constrain(size\_t m, T &)

Return a row vector of specified dimensionality made up of the next scalars.

**Definition:** reader.hpp:266

stan::prob::prob\_constrain

T prob\_constrain(const T x)

Return a probability value constrained to fall between 0 and 1 (inclusive) for the specified free sca...

**Definition:** transform.hpp:875

stan::math::check\_unit\_vector

bool check\_unit\_vector(const char \*function, const Eigen::Matrix< T\_prob, Eigen::Dynamic, 1 > &theta, const char \*name, T\_result \*result)

Return true if the specified vector is unit vector.

**Definition:** check\_unit\_vector.hpp:27

stan::io::reader::matrix\_constrain

matrix\_t matrix\_constrain(size\_t m, size\_t n)

Return a matrix of the specified dimensionality made up of the next scalars arranged in column-major ...

**Definition:** reader.hpp:301

stan::io::reader::unit\_vector

vector\_t unit\_vector(size\_t k)

Return a unit\_vector of the specified size made up of the next scalars.

**Definition:** reader.hpp:768

stan::prob::ub\_constrain

boost::math::tools::promote\_args< T, TU >::type ub\_constrain(const T x, const TU ub)

Return the upper-bounded value for the specified unconstrained scalar and upper bound.

**Definition:** transform.hpp:604

stan::io::reader::row\_vector\_lb\_constrain

row\_vector\_t row\_vector\_lb\_constrain(const TL lb, size\_t m)

**Definition:** reader.hpp:1173

stan::io::reader::matrix\_t

Eigen::Matrix< T, Eigen::Dynamic, Eigen::Dynamic > matrix\_t

**Definition:** reader.hpp:63

stan::io::reader::vector\_ub\_constrain

vector\_t vector\_ub\_constrain(const TU ub, size\_t m, T &lp)

**Definition:** reader.hpp:1227

stan::io::reader::map\_vector\_t

Eigen::Map< vector\_t > map\_vector\_t

**Definition:** reader.hpp:68

stan::io::reader::cholesky\_factor

matrix\_t cholesky\_factor(size\_t M, size\_t N)

Return the next Cholesky factor with the specified dimensionality, reading it directly without transf...

**Definition:** reader.hpp:956

stan::io::reader::ordered

vector\_t ordered(size\_t k)

Return the next vector of specified size containing values in ascending order.

**Definition:** reader.hpp:864

stan::io::reader

A stream-based reader for integer, scalar, vector, matrix and array data types, with Jacobian calcula...

**Definition:** reader.hpp:34

stan::io::reader::reader

reader(std::vector< T > &data\_r, std::vector< int > &data\_i)

Construct a variable reader using the specified vectors as the source of scalar and integer values fo...

**Definition:** reader.hpp:83

stan::io::reader::scalar\_ub\_constrain

T scalar\_ub\_constrain(const TU ub)

Return the next scalar transformed to have the specified upper bound.

**Definition:** reader.hpp:591

stan::io::reader::matrix\_lub\_constrain

matrix\_t matrix\_lub\_constrain(const TL lb, const TU ub, size\_t m, size\_t n, T &lp)

**Definition:** reader.hpp:1343

stan::io::reader::integer\_lub

int integer\_lub(int lb, int ub)

Return the next integer, checking that it is less than or equal to the specified upper bound...

**Definition:** reader.hpp:419

stan::prob::lb\_constrain

T lb\_constrain(const T x, const TL lb)

Return the lower-bounded value for the specified unconstrained input and specified lower bound...

**Definition:** transform.hpp:520

stan::io::reader::matrix

matrix\_t matrix(size\_t m, size\_t n)

Return a matrix of the specified dimensionality made up of the next scalars arranged in column-major ...

**Definition:** reader.hpp:287

stan::math::check\_cholesky\_factor

bool check\_cholesky\_factor(const char \*function, const Eigen::Matrix< T\_y, Eigen::Dynamic, Eigen::Dynamic > &y, const char \*name, T\_result \*result)

Return true if the specified matrix is a valid Cholesky factor.

**Definition:** check\_cholesky\_factor.hpp:30

stan::io::reader::corr\_constrain

T corr\_constrain(T &lp)

Return the next scalar transformed to be a (partial) correlation between -1 and 1, incrementing the specified reference with the log of the absolute Jacobian determinant.

**Definition:** reader.hpp:754

stan::io::reader::row\_vector\_lub\_constrain

row\_vector\_t row\_vector\_lub\_constrain(const TL lb, const TU ub, size\_t m, T &lp)

**Definition:** reader.hpp:1319

stan::io::reader::scalar

T scalar()

Return the next scalar in the sequence.

**Definition:** reader.hpp:155

stan::io::reader::scalar\_pos\_constrain

T scalar\_pos\_constrain(T &lp)

Return the next scalar transformed to be positive, incrementing the specified reference with the log ...

**Definition:** reader.hpp:500

stan::io::reader::ordered\_constrain

vector\_t ordered\_constrain(size\_t k)

Return the next ordered vector of the specified length.

**Definition:** reader.hpp:879

stan::io::reader::matrix\_lb

matrix\_t matrix\_lb(const TL lb, size\_t m, size\_t n)

**Definition:** reader.hpp:1188

stan::io::reader::integer\_lub\_constrain

int integer\_lub\_constrain(int lb, int ub)

Return the next integer, checking that it is less than or equal to the specified upper bound...

**Definition:** reader.hpp:443

stan::io::reader::cholesky\_factor\_constrain

matrix\_t cholesky\_factor\_constrain(size\_t M, size\_t N, T &lp)

Return the next Cholesky factor with the specified dimensionality, reading from an unconstrained vect...

**Definition:** reader.hpp:993

stan::io::reader::prob\_constrain

T prob\_constrain()

Return the next scalar transformed to be a probability between 0 and 1.

**Definition:** reader.hpp:692

stan::io::reader::simplex

vector\_t simplex(size\_t k)

Return a simplex of the specified size made up of the next scalars.

**Definition:** reader.hpp:816

stan::prob::positive\_ordered\_constrain

Eigen::Matrix< T, Eigen::Dynamic, 1 > positive\_ordered\_constrain(const Eigen::Matrix< T, Eigen::Dynamic, 1 > &x)

Return an increasing positive ordered vector derived from the specified free vector.

**Definition:** transform.hpp:1317

stan::prob::unit\_vector\_constrain

Eigen::Matrix< T, Eigen::Dynamic, 1 > unit\_vector\_constrain(const Eigen::Matrix< T, Eigen::Dynamic, 1 > &y)

Return the unit length vector corresponding to the free vector y.

**Definition:** transform.hpp:1015

stan::io::reader::corr\_matrix

matrix\_t corr\_matrix(size\_t k)

Returns the next correlation matrix of the specified dimensionality.

**Definition:** reader.hpp:1107

stan::io::reader::vector\_lb

vector\_t vector\_lb(const TL lb, size\_t m)

**Definition:** reader.hpp:1144

stan::io::reader::scalar\_lb\_constrain

T scalar\_lb\_constrain(const TL lb, T &lp)

Return the next scalar transformed to have the specified lower bound, incrementing the specified refe...

**Definition:** reader.hpp:553

stan::math::check\_positive

bool check\_positive(const char \*function, const T\_y &y, const char \*name, T\_result \*result)

**Definition:** check\_positive.hpp:54

stan::math::check\_corr\_matrix

bool check\_corr\_matrix(const char \*function, const Eigen::Matrix< T\_y, Eigen::Dynamic, Eigen::Dynamic > &y, const char \*name, T\_result \*result)

Return true if the specified matrix is a valid correlation matrix.

**Definition:** check\_corr\_matrix.hpp:38

stan::io::reader::vector\_lb\_constrain

vector\_t vector\_lb\_constrain(const TL lb, size\_t m)

**Definition:** reader.hpp:1151

stan::prob::cov\_matrix\_constrain

Eigen::Matrix< T, Eigen::Dynamic, Eigen::Dynamic > cov\_matrix\_constrain(const Eigen::Matrix< T, Eigen::Dynamic, 1 > &x, typename math::index\_type< Eigen::Matrix< T, Eigen::Dynamic, 1 > >::type K)

Return the symmetric, positive-definite matrix of dimensions K by K resulting from transforming the s...

**Definition:** transform.hpp:1769

stan::io::reader::positive\_ordered

vector\_t positive\_ordered(size\_t k)

Return the next vector of specified size containing positive values in ascending order.

**Definition:** reader.hpp:908

stan::io::reader::available

size\_t available()

Return the number of scalars remaining to be read.

**Definition:** reader.hpp:101

stan::io::reader::cov\_matrix\_constrain

matrix\_t cov\_matrix\_constrain(size\_t k, T &lp)

Return the next covariance matrix of the specified dimensionality, incrementing the specified referen...

**Definition:** reader.hpp:1092

stan::io::reader::row\_vector\_lub\_constrain

row\_vector\_t row\_vector\_lub\_constrain(const TL lb, const TU ub, size\_t m)

**Definition:** reader.hpp:1312

stan::prob::corr\_matrix\_constrain

Eigen::Matrix< T, Eigen::Dynamic, Eigen::Dynamic > corr\_matrix\_constrain(const Eigen::Matrix< T, Eigen::Dynamic, 1 > &x, typename math::index\_type< Eigen::Matrix< T, Eigen::Dynamic, 1 > >::type k)

Return the correlation matrix of the specified dimensionality derived from the specified vector of un...

**Definition:** transform.hpp:1644

stan::io::reader::~reader

~reader()

Destroy this variable reader.

**Definition:** reader.hpp:94

stan::io::reader::row\_vector\_lub

row\_vector\_t row\_vector\_lub(const TL lb, const TU ub, size\_t m)

**Definition:** reader.hpp:1305

stan::io::reader::integer

int integer()

Return the next integer in the integer sequence.

**Definition:** reader.hpp:119

stan::io::reader::row\_vector\_t

Eigen::Matrix< T, 1, Eigen::Dynamic > row\_vector\_t

**Definition:** reader.hpp:65

stan::math::check\_less\_or\_equal

bool check\_less\_or\_equal(const char \*function, const T\_y &y, const T\_high &high, const char \*name, T\_result \*result)

**Definition:** check\_less\_or\_equal.hpp:52

stan::io::reader::matrix\_ub\_constrain

matrix\_t matrix\_ub\_constrain(const TU ub, size\_t m, size\_t n, T &lp)

**Definition:** reader.hpp:1273

transform.hpp

stan::io::reader::matrix\_ub\_constrain

matrix\_t matrix\_ub\_constrain(const TU ub, size\_t m, size\_t n)

**Definition:** reader.hpp:1265

stan::prob::lub\_constrain

boost::math::tools::promote\_args< T, TL, TU >::type lub\_constrain(const T x, TL lb, TU ub)

Return the lower- and upper-bounded scalar derived by transforming the specified free scalar given th...

**Definition:** transform.hpp:710

stan::io::reader::cholesky\_factor\_constrain

matrix\_t cholesky\_factor\_constrain(size\_t M, size\_t N)

Return the next Cholesky factor with the specified dimensionality, reading from an unconstrained vect...

**Definition:** reader.hpp:975

stan::io::reader::integer\_constrain

int integer\_constrain()

Return the next integer in the integer sequence.

**Definition:** reader.hpp:133

stan::io::reader::available\_i

size\_t available\_i()

Return the number of integers remaining to be read.

**Definition:** reader.hpp:110

stan::io::reader::row\_vector\_lb

row\_vector\_t row\_vector\_lb(const TL lb, size\_t m)

**Definition:** reader.hpp:1166

stan::io::reader::vector\_lub\_constrain

vector\_t vector\_lub\_constrain(const TL lb, const TU ub, size\_t m)

**Definition:** reader.hpp:1290

stan::math::check\_positive\_ordered

bool check\_positive\_ordered(const char \*function, const Eigen::Matrix< T\_y, Eigen::Dynamic, 1 > &y, const char \*name, T\_result \*result)

Return true if the specified vector contains only non-negative values and is sorted into increasing o...

**Definition:** check\_positive\_ordered.hpp:29

stan::io::reader::map\_matrix\_t

Eigen::Map< matrix\_t > map\_matrix\_t

**Definition:** reader.hpp:67

stan::io::reader::integer\_lb

int integer\_lb(int lb)

Return the next integer, checking that it is greater than or equal to the specified lower bound...

**Definition:** reader.hpp:331

stan::io::reader::integer\_lub\_constrain

int integer\_lub\_constrain(int lb, int ub, T &)

Return the next integer, checking that it is less than or equal to the specified upper bound...

**Definition:** reader.hpp:457

stan::io::reader::scalar\_ub\_constrain

T scalar\_ub\_constrain(const TU ub, T &lp)

Return the next scalar transformed to have the specified upper bound, incrementing the specified refe...

**Definition:** reader.hpp:607

stan::io::reader::scalar\_lub\_constrain

T scalar\_lub\_constrain(TL lb, TU ub, T &lp)

Return the next scalar transformed to be between the the specified lower and upper bounds...

**Definition:** reader.hpp:665

stan::io::reader::corr\_matrix\_constrain

matrix\_t corr\_matrix\_constrain(size\_t k)

Return the next correlation matrix of the specified dimensionality.

**Definition:** reader.hpp:1123

stan::io::reader::unit\_vector\_constrain

Eigen::Matrix< T, Eigen::Dynamic, 1 > unit\_vector\_constrain(size\_t k)

Return the next unit\_vector transformed vector of the specified length.

**Definition:** reader.hpp:786

stan::prob::positive\_constrain

T positive\_constrain(const T x)

Return the positive value for the specified unconstrained input.

**Definition:** transform.hpp:446

stan::io::reader::matrix\_constrain

matrix\_t matrix\_constrain(size\_t m, size\_t n, T &)

Return a matrix of the specified dimensionality made up of the next scalars arranged in column-major ...

**Definition:** reader.hpp:317

stan::io::reader::vector\_lb\_constrain

vector\_t vector\_lb\_constrain(const TL lb, size\_t m, T &lp)

**Definition:** reader.hpp:1158

stan::io::reader::ordered\_constrain

vector\_t ordered\_constrain(size\_t k, T &lp)

Return the next ordered vector of the specified size, incrementing the specified reference with the l...

**Definition:** reader.hpp:894

stan::io::reader::cholesky\_corr\_constrain

matrix\_t cholesky\_corr\_constrain(size\_t K)

Return the next Cholesky factor for a correlation matrix with the specified dimensionality, reading from an unconstrained vector of the appropriate size.

**Definition:** reader.hpp:1026

stan::prob::cholesky\_corr\_constrain

Eigen::Matrix< T, Eigen::Dynamic, Eigen::Dynamic > cholesky\_corr\_constrain(const Eigen::Matrix< T, Eigen::Dynamic, 1 > &y, int K)

**Definition:** transform.hpp:1512

stan::io::reader::prob

T prob()

Return the next scalar, checking that it is a valid value for a probability, between 0 (inclusive) an...

**Definition:** reader.hpp:677

stan::io::reader::positive\_ordered\_constrain

vector\_t positive\_ordered\_constrain(size\_t k, T &lp)

Return the next positive\_ordered vector of the specified size, incrementing the specified reference w...

**Definition:** reader.hpp:940

stan::io::reader::row\_vector\_ub\_constrain

row\_vector\_t row\_vector\_ub\_constrain(const TU ub, size\_t m, T &lp)

**Definition:** reader.hpp:1249

stan::io::reader::scalar\_lub

T scalar\_lub(const TL lb, const TU ub)

Return the next scalar, checking that it is between the specified lower and upper bound...

**Definition:** reader.hpp:626

stan::io::reader::row\_vector\_lb\_constrain

row\_vector\_t row\_vector\_lb\_constrain(const TL lb, size\_t m, T &lp)

**Definition:** reader.hpp:1180

stan::io::reader::matrix\_lb\_constrain

matrix\_t matrix\_lb\_constrain(const TL lb, size\_t m, size\_t n)

**Definition:** reader.hpp:1196

stan::math::check\_greater\_or\_equal

bool check\_greater\_or\_equal(const char \*function, const T\_y &y, const T\_low &low, const char \*name, T\_result \*result)

**Definition:** check\_greater\_or\_equal.hpp:58

stan::io::reader::vector\_ub\_constrain

vector\_t vector\_ub\_constrain(const TU ub, size\_t m)

**Definition:** reader.hpp:1220

stan::io::reader::simplex\_constrain

Eigen::Matrix< T, Eigen::Dynamic, 1 > simplex\_constrain(size\_t k)

Return the next simplex transformed vector of the specified length.

**Definition:** reader.hpp:834

stan::math::check\_cholesky\_factor\_corr

bool check\_cholesky\_factor\_corr(const char \*function, const Eigen::Matrix< T\_y, Eigen::Dynamic, Eigen::Dynamic > &y, const char \*name, T\_result \*result)

Return true if the specified matrix is a valid Cholesky factor.

**Definition:** check\_cholesky\_factor\_corr.hpp:34

stan::io::reader::vector\_constrain

vector\_t vector\_constrain(size\_t m, T &)

Return a column vector of specified dimensionality made up of the next scalars.

**Definition:** reader.hpp:229


---

[ Stan Home Page ]

*© 2011–2014,
Stan Development Team.*
